# Systemic Chemotherapy prior to Cytoreductive Surgery and HIPEC for Carcinomatosis from Appendix Cancer: Impact on Perioperative Outcomes and Short-Term Survival

**DOI:** 10.1155/2012/163284

**Published:** 2012-07-26

**Authors:** Lana Bijelic, Anjali S. Kumar, O. Anthony Stuart, Paul H. Sugarbaker

**Affiliations:** ^1^Section of Surgical Oncology, Department of Surgery, Washington Hospital Center, 110 Irving Street, Washington, DC 20010, USA; ^2^Section of Colon and Rectal Surgery, Washington Hospital Center, 110 Irving Street, Washington, DC 20010, USA; ^3^Medstar Health Research Institute, Washington Hospital Center, 110 Irving Street, Washington, DC 20010, USA

## Abstract

*Background and Objectives*. Systemic chemotherapy administered prior to cytoreductive surgery and hyperthermic intraperitoneal chemotherapy (HIPEC) for peritoneal mucinous adenocarcinoma of appendiceal origin (PMCA) is associated with a significant rate of histological response. The impact of preoperative systemic chemotherapy (PSC) on intraperitoneal tumor burden, completeness of cytoreduction, and perioperative complications is unknown. *Methods*. We analyzed prospectively collected data from our HIPEC database. Thirty-four patients with PMCA were prospectively recruited and treated with PSC. Perioperative variables and survival in this group of patients were compared against 24 patients with PMCA who did not receive PSC. *Results*. Ten of 34 patients (29%) receiving PSC had a complete or near complete histological response. Patients receiving PSC had a lower peritoneal carcinomatosis index, required fewer peritonectomies and visceral resections, and achieved complete cytoreduction more frequently compared to patients with no preoperative chemotherapy. The incidence of perioperative complications and survival were not significantly different between the two groups. However, patients with complete histological response had better overall survival compared to patients without complete response. *Conclusions*. Preoperative systemic chemotherapy in appendix-originated PMCA is associated with a significant rate of histological response which may reduce the tumor burden, facilitate less aggressive and more complete CRS, and improve short-term survival in patients with a significant histological response.

## 1. Background

The use of neoadjuvant systemic chemotherapy prior to surgery for a primary, usually locally advanced, or metastatic malignancy has been extensively studied. The potential advantages of neoadjuvant chemotherapy include a reduction of tumor volume with a greater chance for complete surgical removal and organ preservation. The role of neoadjuvant chemotherapy or chemoradiation is well established in breast, rectal, and to a lesser extent, esophageal and ovarian cancers [1–4]. It has also been explored in association with surgery for liver metastases from colon cancer [5]. Although this approach may have important advantages in terms of improving resectability and local control, it generally does not improve overall survival when compared to adjuvant chemotherapy [2]. 

The benefit of neoadjuvant therapy may be more apparent among mucinous appendiceal neoplasms, which are often associated with peritoneal involvement at the time of diagnosis. The optimal treatment for this condition involves cytoreductive surgery and heated intraperitoneal chemotherapy (HIPEC) which results in long-term survival ranging from 30–80% at 20 years [6–8]. The histological characteristics of the peritoneal metastases range from adenomucinosis which has an excellent long-term outcome when treated with cytoreductive surgery and HIPEC to peritoneal mucinous adenocarcinoma (PMCA) which has a less favorable outcome despite this aggressive treatment [9]. Therefore, systemic therapy as an adjunct to cytoreductive surgery and HIPEC for PMCA is often utilized based on the effectiveness of FOLFOX chemotherapy in advanced colon cancer [10]. Recently, there have been studies indicating reasonable activity of several 5-FU-based regimens in patients with advanced carcinomatosis from appendix cancer [11]. We have previously published our initial experience using FOLFOX chemotherapy as a neoadjuvant treatment prior to cytoreductive surgery and HIPEC in 34 patient with PMCA [12]. This initial manuscript described the clinical and histological parameters of response and showed that the ability of clinical examination and CT imaging to assess response to treatment was limited. However, pathologic examination showed a significant histological response in almost 30% of patients. In this study, we report the impact of neoadjuvant chemotherapy on perioperative outcomes including extent of cytoreductive surgery and morbidity as well as early survival in the original cohort of 34 patients and in a comparison group of 24 patients who were not treated with neoadjuvant chemotherapy.

## 2. Methods

Patients with histologically confirmed PMCA of appendiceal origin treated with cytoreductive surgery and HIPEC at the Washington Cancer Institute between January 2005 and December 2009 were retrospectively identified from a prospectively-collected database. Permission to collect and analyze this data was obtained from our Institutional Review Board.

From January 2005 until July 2009, patients with PMCA who were thought to be candidates for cytoreductive surgery and HIPEC at the time of their referral were enrolled in a prospective clinical pathway and treated with systemic chemotherapy prior to cytoreductive surgery. All of these patients had the diagnosis confirmed histologically at the time of initial laparotomy or laparoscopy and their slides reviewed to confirm the diagnosis of PMCA. Systemic chemotherapy consisted of a 5-FU- or capecitabine-based regimen with oxaliplatin. The choice of the specific regimen and the use of bevacizumab were at the discretion of the treating medical oncologist. The recommended initial duration of the therapy was 6 cycles followed by imaging and clinical evaluation. Additional 6 cycles of therapy were permitted if there was no evidence of progression at the completion of the first 6 cycles. Following completion of systemic chemotherapy, all patients underwent cytoreductive surgery and HIPEC. During this time period, 22 patients did not have systemic chemotherapy prior to cytoreductive surgery due to their refusal to participate in the prospective clinical pathway or inability to appropriately coordinate treatment with oncologists outside our institution and instead were treated with CRS and HIPEC upfront. Following the completion of our prospective observational study evaluating the use of routine preoperative systemic chemotherapy prior to CRS in July 2009, patients undergoing CRS did not receive routine systemic chemotherapy prior to surgery. Therefore, from July 2009 until December 2009 2 additional patients who did not receive systemic chemotherapy prior to CRS and HIPEC were identified in our database. All of these patients constituted the control group of 24 patients without neoadjuvant systemic chemotherapy in this analysis. Patients who were referred to our center after receiving multiple lines of systemic chemotherapy or who were treated with systemic chemotherapy because they were thought to have unresectable disease on initial evaluation were excluded from this analysis.

Cytoreductive surgery was performed by the senior author in all cases and consisted of peritonectomies and visceral resections performed as needed to achieve complete tumor removal whenever possible as previously described [13]. After all resections were completed, the patients underwent HIPEC for 90 minutes. The HIPEC regimen consisted of mitomycin C and doxorubicin at 15 mg/m^2^ administered intraperitoneally at 42°C with simultaneous infusion of 5-FU 400 mg/m^2^ and leucovorin 20 mg/m^2^ intravenously. Early postoperative intraperitoneal chemotherapy with 5-FU was used selectively in patients who did not have more than 6 cycles of preoperative systemic chemotherapy and/or who had a moderate cytoreduction without multiple or high risk intestinal anastomoses. Perioperative variables including the peritoneal cancer index, completeness of cytoreduction, and a detailed assessment of morbidity by grade and organ system for each patient were prospectively assessed and entered into our database. For patients treated with neoadjuvant chemotherapy, response was assessed histologically by comparing the microscopic characteristics of the tumor resected at the time of cytoreduction to the appearance at the time of the initial diagnosis. A histological near-complete response was defined as the presence of adenomucinosis alone or the presence of extensive fibrosis with only sporadic malignant epithelial cells. A histological complete response was defined as absence of any tumor seen despite extensive sampling at the time of CRS.

## 3. Results

There were a total of 58 patients with PMCA identified in our HIPEC database during the study period: 34 patients who received systemic chemotherapy prior to CRS and HIPEC and 24 who did not. There were 27 males and 31 females with a mean age of 50.7 years. There were no differences between the 2 groups in terms of gender, age, histology, or lymph node status. The demographic and systemic chemotherapy data on the 34 patients who received and the 24 patients who did not receive systemic chemotherapy prior to cytoreductive surgery is shown in Table 1.

For the 34 patients treated with neoadjuvant systemic chemotherapy, none of the analyzed clinical factors including histological subtype, presence of positive lymph nodes, type of systemic regimen used, duration of preoperative systemic chemotherapy, or use of bevacizumab were predictive of histological complete or near-complete response.

In Table 2, the data gathered perioperatively in the 34 patients treated with neoadjuvant chemotherapy was statistically compared to the 24 patients who did not receive neoadjuvant chemotherapy prior to CRS and HIPEC. Patients receiving preoperative systemic chemotherapy had a lower peritoneal carcinomatosis index (mean 19) compared to patients not receiving neoadjuvant chemotherapy (mean 28, *P* = 0.0003). The mean number of peritonectomies (2.3 versus 3.7) and visceral resections (2.7 versus 4.4) was also significantly lower in patients who received preoperative systemic chemotherapy. Twenty-six of the 34 patients treated with neoadjuvant chemotherapy had grade 3 or 4 complications following cytoreductive surgery, similar to patients not treated with preoperative chemotherapy (14 of 24 patients, *P* = 0.16).

**Figure 1:**
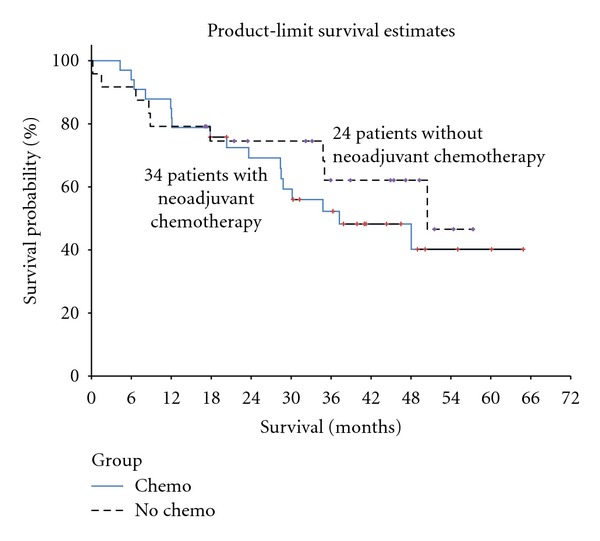
Kaplan-Meier survival analysis of 34 patients with PMCA from appendix cancer treated with neoadjuvant chemotherapy (median survival 37.2 months) compared to 24 patients who did not receive neoadjuvant chemotherapy prior cytoreduction and HIPEC (median survival 50.5 months). The difference in survival is not significant (*P* = 0.56).

**Figure 2:**
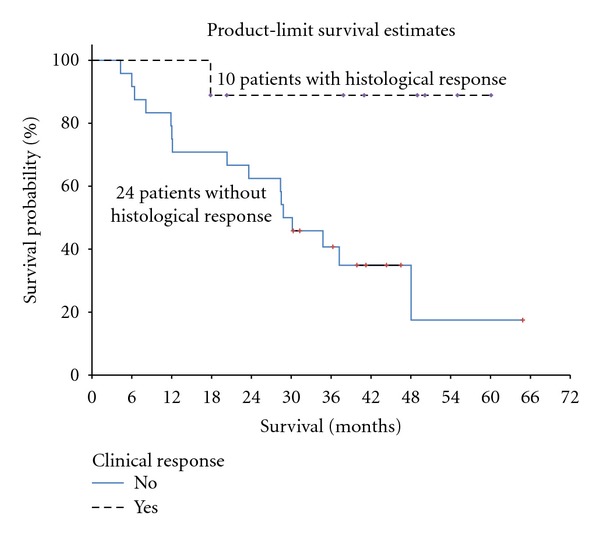
Kaplan-Meier survival analysis of 10 patients with PMCA from appendix cancer treated with neoadjuvant systemic chemotherapy prior to cytoreductive surgery and HIPEC who had a complete or near-complete histological response (median survival not reached) compared to 24 patients who had no significant histological response (median survival 29.5 months). The difference is statistically significant (*P* = 0.032).

Median survival for patients receiving neoadjuvant chemotherapy was 37.2 months compared to 50.5 months for patients who did not receive preoperative chemotherapy (*P* = 0.56, Figure 1). However, among the patients who received neoadjuvant chemotherapy, survival was significantly better for patients who experienced a histological complete or near complete response (median survival not reached compared to patients with no histological response (median survival 29.5 months, *P* = 0.033, Figure 2)).

## 4. Discussion

We have previously documented that a substantial number of patients in our prospective cohort of PMCA patients treated with neoadjuvant systemic chemotherapywill have a favorable histological response [12]. This may be seen as a transition of PMCA into adenomucinosis, a marked fibrosis with only scattered malignant cells seen or a complete absence of any cancer cells. Having the ability to predict such significant response based on available clinical factors would improve our ability to select the appropriate patients for this treatment and guide certain aspects of the treatment. However, our data showed that none of the analyzed factors could predict response. This is similar to other experiences with preoperative chemotherapy or chemoradiation where clinical factors or imaging studies often fail to accurately predict pathological response [14, 15]. Some studies have used microarray analysis and genomic analysis to improve the ability to predict response to therapy in rectal and breast cancer. Unfortunately, to date, similar studies are not available in patients with appendiceal cancers [16, 17].

Another potential advantage of neoadjuvant chemotherapy is a reduction in tumor volume which can sometime translate into less extensive surgical procedures and allow for improved organ preservation. This is well established in breast cancer where the use of neoadjuvant chemotherapy in patients with locally advanced disease can offer the possibility of breast conservation in a significant number of patients who would otherwise require a mastectomy [18, 19]. Similarly, the use of neoadjuvant chemoradiation in rectal cancer was shown to be associated with an increased number of sphincter preserving procedures in a large prospective study [20]. The assessment of tumor volume and of the impact that a decrease in tumor burden may have on the surgery that is performed is more difficult in patients with peritoneal metastases because both clinical exam and imaging evaluations are inaccurate in assessing the extent of disease. The extent of the peritonectomies and visceral resection required for complete cytoreduction at best can only be estimated based on preoperative imaging. The final decision making is done at the time of the surgical exploration. Therefore, we attempted to evaluate whether neoadjuvant chemotherapy had an impact on the tumor burden and the extent of cytoreductive surgery by comparing 34 patients who received neoadjuvant chemotherapy to a cohort of 24 patients with PMCA who received cytoreductive surgery first. Both groups received the same HIPEC regimen. We found a significantly lower PCI in patients treated with neoadjuvant chemotherapy suggesting a decrease in tumor burden (downsizing). As could be expected, this translated into a less extensive surgical procedure: the number of peritonectomies and the number of visceral resections were lower in the group of patients treated with neoadjuvant chemotherapy. A similar observation was made when neoadjuvant chemotherapy was studied in patients with advanced ovarian cancer: a significantly larger number of patients who received neoadjuvant chemotherapy was able to undergo complete cytoreduction compared to patients who had primary debulking surgery [21]. These results seem to suggest a significant advantage for the use of neoadjuvant chemotherapy but must be interpreted with some caution considering the nonrandomized nature of our study. Another potential problem in the use of neoadjuvant chemotherapy for peritoneal surface malignancies concerns some difficulties in assessing the gross findings inthe operating room. This may lead to incomplete cytoreduction in patients whose frozen section analysis fails to demonstrate residual tumor that is later confirmed on immunohistochemistry or in cases of severe posttreatment fibrosis which makes peritonectomy impossible. However, despite a lower PCI and less extensive cytoreductive surgery, the rate of grade 3 and 4 complications was not significantly different in the two groups. 

The impact of neoadjuvant chemotherapy on survival and locoregional control has been studied extensively in breast, rectal, esophageal, and other cancers. Although early reports have sometimes suggested a survival advantage for neoadjuvant therapy, this is usually lost with longer followup. More definitive randomized studies have shown that there is no survival advantage of neoadjuvant compared to adjuvant chemotherapy in breast cancer [2, 18, 19]. In rectal cancer, there is a benefit of preoperative chemoradiation in local control but no survival advantage. The results of a recent randomized study evaluating the role of neoadjuvant chemotherapy in patients with advanced ovarian cancer showed equivalent overall and progression-free survival for patients treated with primary debulking surgery and those treated with neoadjuvant chemotherapy followed by debulking surgery [20–22]. Our study is in agreement with these observations. In this experience, there is no improvement in overall survival in patients treated with neoadjuvant chemotherapy compared to patients without neoadjuvant treatment, but it is important to observe that adjuvant systemic therapy was recommended for these patients. However, considering the high rate of histological complete or near complete response in our cohort of patients, it would be important to know if this subset of patients has an improved survival. Indeed, our early data suggests that the patients who have a histologically significant response have a better short-term survival than those who do not have a significant response. It will be important to follow these patients in the future to determine whether this survival advantage will persist with longer followup. The fact that only patients with histologically significant response seem to have an improvement in survival at least in the short-term further emphasizes the importance of developing clinically useful predictors of response.

In summary, our experience suggests that 6 cycles of systemic chemotherapy prior to cytoreductive surgery for PMCA from appendix cancer may be associated with a reduction in tumor burden which may facilitate a less extensive cytoreductive procedure. We did not observe a significant change in postoperative complications in this group of patients compared to patients who were not treated with preoperative chemotherapy. Although the group as a whole does not seem to have an improved survival compared to patients with PMCA who receive systemic chemotherapy following CRS and HIPEC, the subgroup of patients with complete or near complete histological response appears to have better short-term survival compared to the group of patients without a histological response to preoperative chemotherapy.

## Figures and Tables

**Table 1:** Demographic and treatment data on 34 patients with PMCA from appendix cancer who received neoadjuvant systemic chemotherapy prior to cytoreductive surgery and HIPEC. None of these clinical parameters were predictive of histological response.

	Patients treated with neoadjuvant chemotherapy	Patients not treated with neoadjuvant chemotherapy	*P* value
Age (mean)	47.9	48.8	0.66
Gender		0.53
Male	17	10	
Female	17	14	
Histological subtype		0.37
Signet ring	9	4	
PMCA/adenocarcinoid	25	20	
Lymph node status		0.62
Positive	12	7	
Negative	22	17	
Number of preoperative chemotherapy cycles	N/A	
6 cycles	12		
12 cycles	22		
Chemotherapy regimens	N/A	
FOLFOX	30		
XELOX	4		
Use of bevacizumab	N/A	
Yes	21		
No	13		
Gross assessment of response at cytoreduction	N/A	
Stable or response	16		
Progression	17		
Histological assessment of response		
No response	24		
Complete or near-complete response	10		

**Table 2:** Comparison of perioperative variables between 34 patients treated with neoadjuvant chemotherapy prior to cytoreductive surgery and HIPEC and 24 patients that did not receive preoperative chemotherapy before cytoreductive surgery and HIPEC.

Clinical characteristic	Patients with PMCA from appendix cancer		
	Neoadjuvant chemotherapy	No neoadjuvant chemotherapy	*P* value
Number of patients	34	24	
Peritoneal cancer index (mean)	19	28	0.0003
Number of peritonectomies	0.0032		
Mean	2.3	3.7	
Range	0–5	1–5
Number of visceral resections	<0.001		
Mean	2.7	4.4
Range	1–5	2–7
Completeness of cytoreduction	0.78		
CCR 0/CCR 1	22	12
CCR 2	7	5
CCR 3	5	5	
Complications	0.16		
None or grade1/2	8	10	
Grade 3 or 4	26	14	
